# The impact of the English calorie labelling policy on the energy content of food offered and purchased in worksite cafeterias: a natural experiment

**DOI:** 10.1186/s40795-024-00914-1

**Published:** 2024-08-13

**Authors:** Madison Luick, Lauren Bandy, Susan A. Jebb, Rachel Pechey

**Affiliations:** Nuffield Department of Primary Care Health Sciences, University of Oxford, Radcliffe Primary Care Building, Radcliffe Observatory Quarter, Woodstock Rd, Oxford, OX2 6GG UK

**Keywords:** Calorie labelling, Policy, Energy, Food, Worksite cafeterias

## Abstract

**Background:**

On 6 April 2022, legislation came into effect in England requiring calorie labels to be applied to food items on menus of larger food businesses. This study aimed to assess the impact of calorie labelling on (a) food purchased and (b) energy content of menu options in worksite cafeterias.

**Methods:**

Product-level sales data and energy content of available items was obtained from 142 worksite cafeterias from January 2022-October 2022. Interrupted-time-series (ITS) analysis with level and slope change evaluated daily energy (kcal) purchased per item, and ITS with level change assessed mean energy per option available on menus before and after calorie labelling. Each analysis was conducted 6 weeks and 6 months from implementation. A post-hoc ITS examined weekly energy purchased per item over a longer period (March 2021-October 2022; 135 sites).

**Results:**

There was no evidence calorie labelling changed the energy content of foods purchased (6-week: + 0.60 cal/product, 95%CI:-2.54, + 3.75; 6-month: + 1.59 cal/product, 95%CI:-0.96, + 4.16). Post-hoc analyses suggested calorie labels were associated with a reduction in mean energy of items purchased over time (-0.65 kcal/week, 95%CI:-0.81,-0.49), but a significant increase (+ 3 kcal, 95%CI: + 0.43, + 5.60) at the point of implementation. There was a reduction in the mean energy content of menu options at each seasonal menu change (April 2022:-1.79 kcal, 95%CI:-3.42,-0.15; July 2022:-4.18 kcal, 95% CI:-7.65,-0.73).

**Conclusion:**

This large observational study in worksite cafeterias found no evidence to indicate the introduction of calorie labelling led to any immediate reduction in energy purchased by customers. There was some evidence of increasing impact over time, possibly associated with changes in menu offerings, but this effect was small and cannot be directly attributed to calorie labelling.

**Supplementary Information:**

The online version contains supplementary material available at 10.1186/s40795-024-00914-1.

## Background

Diet related non-communicable diseases, such as obesity, overweight and heart disease, are population health challenges [6]. Calorie labelling has been suggested as a useful policy tool to help individuals better understand the energy content of the food they consume and to become more aware of the food choices they make [7]. However, the actual impact calorie labels have on energy intake is less clear.

Systematic reviews and meta-analyses on the effectiveness of calorie labelling have shown either small reductions of energy purchased or no overall effect [1, 2, 5, 12]. Subsequently, randomised controlled trials across worksite locations in the UK have found no or minimal effects from introducing calorie labels [25, 26]. However, there is some suggestion that the introduction of calorie labels results in a reformulation of menu offerings towards lower energy options [1, 19].

A limitation of calorie labelling studies to date is the difficulty in implementing randomised controlled trials in naturalistic settings with realistic labelling, rather than ‘experimental’ labels that may be more prominent than can be achieved in routine practice. Due to feasibility of the monitoring a randomised controlled trial, many of these studies [4, 9, 10, 25, 26] have implemented calorie labels for a relatively short period of time and at a limited number of sites. This has implications for the statistical power to detect an effect and could possibly contribute to the pattern of null or mixed research findings around the impacts of calorie labels. Yet, food energy labels have been implemented through local and national policies in various settings, and there has been found to be a reduction in energy purchased from such policies [28]. In New South Wales, Australia, the state government introduced kilojoule labels in February 2011, with an early report indicating the labels resulted in a reduction of reported daily kilojoule consumption and improved knowledge of the recommended daily intake [13]. However, there was no evidence that the introduction of labels in New South Wales resulted in the reformulation of menu items to offer lower energy options when five of the largest fast-food chains were analysed [27]. In the United States, where calorie labels have also been introduced nationwide as part of a federal policy, there was observed to be a reduction in energy purchased per transaction, and findings suggested that new menu items introduced may have had reduced energy content [16, 17].

On 6 April 2022, legislation from the UK Government’s Department of Health and Social Care came into effect in England, requiring restaurants, cafes, and takeaways with at least 250 employees to apply calorie labels to their food items [7]. While calorie labelling policies have been assessed in other global settings, to our knowledge, few if any studies have assessed the impact of the calorie labelling policy in England on either consumer behaviour (i.e. food purchased) or business behaviour (i.e. food offered on menus). This study analysed if the introduction of calorie labelling in April 2022 led to changes in either the energy content of food purchased or supplied (i.e. offered on menus) in a large sample of worksite cafeterias up to six months post-implementation.

## Methods

### Data and Study Design

This observational field study utilises data from 142 worksite cafeterias run by a single commercial partner in the UK. Data was available from cafeteria sites the commercial partner runs across Great Britain. The worksites were located in England, Wales, and Scotland. Although the legislation did not make labelling mandatory in Scotland or Wales, the catering provider implemented calorie labels uniformly across their cafeteria sites. The catering provider operates cafeterias across a variety of worksites, with approximately 40% of these being manufacturing or distribution centres, ~40% at administrative or managerial offices, and the remaining ~20% classified as a mix of these two. Each cafeteria site has a base menu provided by the central office, from which they can select the foods which will be served in their specific cafeteria, meaning that while most cafeterias will offer the same basic items (e.g. burgers), they are able to choose which burgers they order and offer. This base menu changes every 12 weeks, and cafeterias run menus on a 4-week cycle (i.e. every four weeks the cafeteria cycles back through the same food items). Cafeterias select the foods they will serve during the 12-week time period from the base menu (but do not typically serve all items from this menu), and repeat this in three 4-week menu cycles.

The primary analysis covers a 3-month period before and 6-month period after the effective date of the new law to compare purchasing activity before and after the implementation, covering January to October 2022. Due to logistical constraints and the legal requirement to universally implement calorie labelling in the UK as of 6 April, it was not possible to randomise study sites or control the rollout of the implementation. This means all worksite cafeterias in the study sample introduced calorie labels at the same time and there could be no control group, given the legal requirement to implement labels.

We analysed data six weeks and six months following the implementation of the policy to assess any changes over time. Additionally, a post-hoc analysis used data from one year before labels were implemented to six months after, to provide more information on seasonal changes.

### Data sources

Sales data from worksite cafeterias were obtained from the commercial partner. Data included product-level sales data (i.e., number of units sold of each item) for selected cafeterias (those who sold a mean of at least 50 meals per day before labels were introduced in April 2022). Individual customer transactions were not available.

Energy labelling was implemented on all products made on site. Products prepared on site were grouped into one of 10 categories for analysis, matching categories within the catering provider system. These categories were: Breakfast; Cakes, Pastries, Biscuits, and Discretionary; Fruits and Vegetables; Jacket Potatoes; Meals; Miscellaneous and Condiments; Salads and Cold Snacks; Sandwiches; Savoury Snacks; and Starters. Energy content of these items during this study period was provided by the commercial partner, as was the cost of each item. Energy data for pre-packaged items (e.g., bottled drinks, pre-packaged crisps and confectionery) were not available from the commercial partner – however, the provision of calorie information for these items did not change as a result of this legislation.

### Study period: primary analysis

The overall study period for primary analysis is from 10 January 2022 to 10 October 2022, with the calorie labels implemented in sites on 4 April 2022. Following the pre-registered analysis plan (https://osf.io/t5jbh/), interrupted time series models were used to assess the potential impact of calorie label implementation. For the interrupted time series assessing level and slope change, the intervention point was at 4 April 2022, with a slope change after this point. For the interrupted time series assessing level changes at the time of catering provider menu changes, time was a fixed effect and menu changes were included as dummy variables. One of the menu changes occurred on 4 April 2022. All models were linear regression models, and for both outcomes, four models were run (using data from six weeks post-labelling, data from six months post-labelling, applying a secondary model, and a post-hoc analysis model). All models are centred at zero (i.e. time 0 represents the start of the intervention), and they are outlined in the below sections and Supplementary File A. Due to evidence of autocorrelation, all ITS and regression models applied Newey-West robust standard errors, with a lag of 28 days for the monthly menu cycles. Data cleaning and statistical analyses were conducted in R version 4.3.2.

### Statistical analysis

#### Impact of calorie labelling implementation on energy purchased

Interrupted time series analysis with level and slope change was used to evaluate the impact of the implementation on energy (kcal) purchased from items made on site before and after the calorie labelling intervention. The primary outcome variable was mean daily calories purchased per food item from aggregated data. Weekends and bank holidays were removed, given the likelihood of unusual trading on these dates. Annual seasonality was difficult to account for, given the study period from January to October, however, a sensitivity analysis was run considering seasonal menu changes as covariates (which occurred approximately every 12 weeks). The primary model can be found in Supplementary File A.

Secondary analyses repeated the same analysis for different food categories (e.g. main meals, sandwiches), to examine the potential for different impacts by category.

Sales data for retail items (i.e. pre-packaged crisps, chocolates or snacks) and drinks were also analysed in secondary analyses for changes in quantity sales, assessing any potential knock-on effects of implementing the calorie labels. In these analyses, the outcome variable was number of items sold.

#### Impact of calorie labelling implementation on menu composition

Interrupted time series analysis with only level change was used to evaluate the impact on mean energy (kcal) of prepared menu items (e.g. hot meals, jacket potato toppings, etc.) offered before and after the implementation of calorie labels. The level-only ITS analysis considered the difference in energy offered on menus, using time as a fixed effect in the linear regression model and including when the catering provider made menu changes during the study period as dummy variables. This model was selected a priori, since it was known prior to the analysis that the menu change would coincide with the implementation of calorie labels on menus, and that another menu change would follow this 12 weeks later, providing the next notable opportunity for a reformulation of the menu. The model used to assess the impact of calorie labelling implementation on menu composition can be found in Supplementary File A.

Secondary analyses repeated the analysis by food category, and a sensitivity analysis considered the overall trend in energy offered, applying an interrupted time series model with level and slope changes. This model was considered, given the possibility that individual catering managers may select different foods from the base menu during the 12-week menu cycle, and this model could better assess a gradual shift over time. The model for the secondary analyses was the same as the primary analysis, and the model for the sensitivity analysis was the same as the energy purchased primary analysis.

### Post-hoc analysis

Following primary analysis, a post-hoc analysis was run, where weekly-level product sales data from March 2021 to October 2022 were obtained. This allowed for a longer period of analysis, with an entire year of data prior to the implementation of calorie labels, so that each week post-implementation had a corresponding week in the pre-implementation stage to better account for seasonal changes. This period of time involved site closures, so there are a variable number of sites open in any given week for the aggregation and analysis, however, to consider this, a sensitivity analysis was also run including only those sites that were in the dataset from the beginning.

An interrupted time series analysis with level and slope change was run for both energy purchased and energy offered. Seasonality was included as a variable for every four-week period, equating to thirteen dummy variables in a fifty-two week-long year. No other additional variables were included (i.e. seasonal menu changes that are included in the primary analysis, since the seasonal week variable accounts for seasonality). Weeks with holidays or with incomplete data were excluded from analysis. The model used for the post-hoc analysis can be found in Supplementary File A.

The same method for robust standard errors was used as in other analyses, applying Newey-West standard errors with a lag of 4 weeks for the monthly menu cycle.

### Sensitivity analysis

Not all sites were in the data set from the beginning to the end of the available data. Sensitivity analyses were run both for the primary analysis and post-hoc analysis models, including only those sites that appeared in the dataset at the first available data point. This included 137 of 142 sites that were in the dataset from the beginning for the primary analysis, and 97 of 142 sites for the post-hoc analysis.

### Deviations from protocol

This analysis was originally intended to be on a dataset from January to September 2022. Additional data was used for a post-hoc analysis in light of concerns about the potential impacts of seasonality on the primary outcome. Secondary analyses on retail products were added in as well, to assess any potential knock-on effects to food items not affected by the change in calorie labelling. The catering provider predominantly offers two different sets of menus, with the menu type often associated with the type of worksite (e.g. manufacturing/distribution vs. office-based); however, information on type of menu offered was not available, so planned analyses exploring whether any impact differed by menu type could not be run. The sensitivity analysis with a reduced number of sites, only including those that were in the dataset from the beginning, was also not originally included in the analysis plan.

## Results

Data was obtained from 142 worksite cafeterias, including both office-based and manufacturing or distribution workplaces, located throughout Great Britain. There were 2371 different foods available with calorie labels, across 10 food categories (breakfast; cakes, pastries, biscuits, and discretionary; fruits and vegetables; jacket potatoes; meals; miscellaneous and condiments; salads and cold snacks; sandwiches; savoury snacks; starter).

Descriptive statistics for primary outcomes by food category are shown in Table 1. For both mean energy purchased and offered, differences in pre- and post-implementation means were observed for: Breakfast, Jacket Potatoes, Miscellaneous and Condiments, Salads and Cold Snacks, and Sandwiches.

**Table 1:** Descriptive statistics for outcome variables in the primary analysis dataset

	Pre-implementation		Post-implementation		Comparison	
	Mean energy per product purchased (mean, SD)	Mean energy per product offered (mean, SD	Mean energy per product purchased (mean, SD)	Mean energy per product offered (mean, SD	*P*-value (energy per product purchased)	*P*-value (energy per product offered)
All food	233 (15)	170 (108)	229 (14)	167 (107)	0.100	0.815
Breakfast	149 (13)	183 (2)	144 (11)	176 (4)	0.002	< 0.0001
Cakes, Pastries, Biscuits, and Discretionary	348 (11)	337 (4)	347 (11)	337 (4)	0.524	0.518
Fruits and Vegetables	265 (9)	206 (11)	268 (12)	203 (9)	0.121	0.090
Jacket Potatoes	225 (7)	199 (4)	229 (8)	201 (4)	0.0002	0.010
Meals	496 (68)	491 (36)	489 (67)	480 (38)	0.463	0.075
Miscellaneous and Condiments	100 (8)	110 (6)	96 (8)	107 (6)	0.005	0.001
Salads and Cold Snacks	154 (9)	156 (6)	150 (9)	151 (5)	0.0006	< 0.0001
Sandwiches	437 (8)	433 (4)	430 (9)	431 (4)	< 0.0001	0.0001
Savoury Snacks	448 (29)	462 (17)	444 (30)	466 (16)	0.293	0.098
Starters	112 (11)	116 (6)	111 (11)	115 (7)	0.425	0.195

Mean values are calculated using the daily aggregate value for sites, and *p*-value is a t-test between pre-implementation and post-implementation means

### Energy purchased

**Fig. 1:**
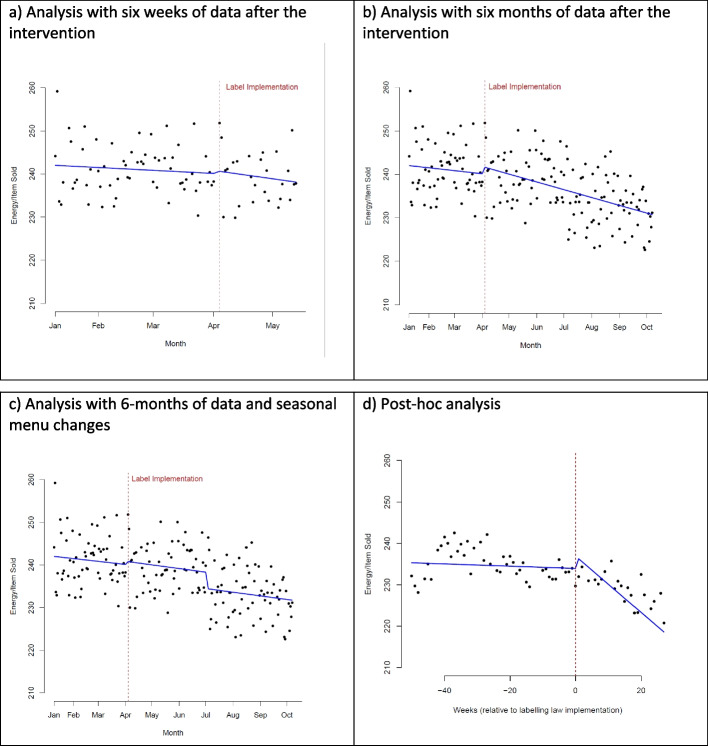
ITS analyses showing average energy per item sold at sites before and after the calorie labelling implementation, showing (**a**) 6 weeks of daily-level data after implementation, (**b**) 6 months of daily-level data after implementation, (**c**) 6 months of daily-level data after implementation and accounting for seasonal menu changes, and (**d**) post-hoc analysis using weekly-level data from a year before and 6 months after implementation. The post-hoc analysis includes a dummy variable for monthly seasonality, however, the graph shown here has these level changes removed (i.e., using the coefficients and output from the model with month dummy variables, but keeping the value for month constant) to better identify the change in trends before and after implementation

There was no evidence that the implementation of calorie labels was associated with a decrease in energy purchased (6-week: + 0.60 kcal/item, 95%CI: -2.54, + 3.75; 6-month: + 1.59, 95%CI: -0.96, + 4.16) (Fig. 1a, b).

This conclusion was unchanged in sensitivity analyses using daily data that included seasonal menu changes (+ 0.73 kcal/item, 95%CI: -1.66, + 3.11). However, from July 2022 (the time of the menu change, 12 weeks after the introduction of calorie labelling) there was a significant decrease in energy purchased (-3.84 kcal/item, 95%CI: -6.35, -1.32) (Fig. 1c).

In the post-hoc analysis, using weekly-level data from one year before to six months after calorie labels were implemented, energy per item purchased increased at the time of the implementation (+ 3 kcal/item, 95%CI: + 0.4, + 5.6), but thereafter decreased over time (-0.65 kcal/item/week, 95%CI: -0.81, -0.49) (Fig. 1d). See Supplementary Tables 1–3 for full interrupted time series models for energy per item purchased.

### Calorie content of options offered

**Fig. 2:**
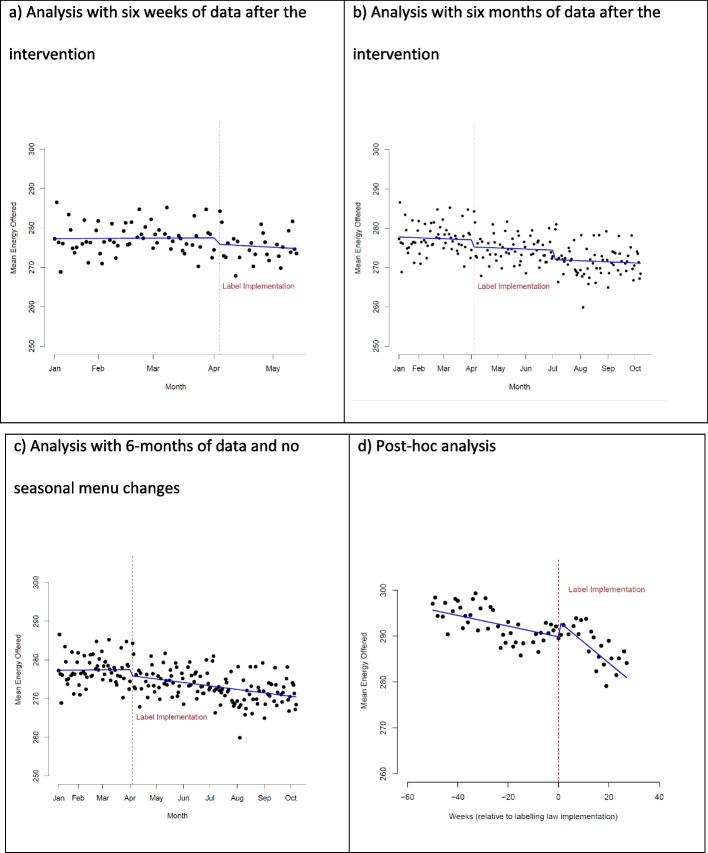
ITS analysis of mean energy offered per item, for periods of analysis including: (**a**) 6 weeks of daily-level data after implementation with menu changes, (**b**) 6 months of daily-level data after implementation with menu changes (including one at the implementation point), (**c**) 6 months of daily-level data after implementation with no menu changes but an implementation point, and (**d**) post-hoc analysis with weekly-level data from a year before and 6 months after implementation. The post-hoc analysis includes a dummy variable for monthly seasonality, however, the graph shown here has these level changes removed (i.e., using the coefficients and output from the model with month dummy variables, but keeping the value for month constant) to better identify the change in trends before and after implementation

Analysis of daily-level data showed the mean energy content of items on menus did not change during the first six weeks of implementation (-2.03 kcal/item, 95%CI: -4.25, + 0.20). However, longer-term analysis showed a decrease following both the April 2022 (-1.79 kcal/item, 95% CI: -3.42, -0.15) and July 2022 (-4.18 kcal/item, 95% CI: -7.65, -0.73) menu changes (Fig. 2a, b).

Due to the potential for seasonality influencing offerings, and since these menu changes included summer months, an additional ITS analysis was run, considering overall trends after implementation. A downward level change was observed at the point of implementation when 6 months of daily-level data after the implementation was considered without accounting for menu changes (6-week analysis: -1.61 kcal/item at point of implementation, 95%CI: -4.25, + 1.04; 6-month analysis: -1.62 kcal/item at point of implementation, 95%CI: -3.19, -0.06) (Fig. 2c).

In the post-hoc analysis, using weekly-level data over an extended time period, there was evidence of a step increase in calories offered at the time of the implementation (+ 3 kcal/item, 1.3 – 5.5). This was followed by a downward trend over time (-0.34 kcal/item/week, 95%CI: -0.48, -0.20) (Fig. 2d). See Supplementary Tables 4–6 for full regression outputs for mean energy per item offered.

### Sensitivity analyses

Sensitivity analyses run for the primary analysis (137 sites), as well as for the post-hoc analysis (97 sites) with only those sites which appeared throughout the dataset were observed to have generally the same pattern of results (see Supplementary Tables 3, 6).

### Secondary analyses

Analysis by food category showed mixed effects. There was a significant decrease in the energy content of breakfast items purchased (6-week: -2.40 kcal/item, 95% CI: -4.24, -0.56; 6-month: -3.90, 95%CI: -5.89, -1.92) and sandwiches (6-week: -6.78 kcal/item, 95%CI: -9.16, -4.39; 6-month: -6.03, 95%CI: -8.19, -3.87) and an increase at one or both time points for other categories; Jacket Potatoes (6-week: 4.52 kcal/item, 95%CI: 3.29, 5.77; 6-month: 2.35, 95%CI: 0.31, 4.39), Fruits and Vegetables (6-week: 9.02 kcal/item, 95%CI: 5.98, 12.06; 6-month: 5.33, 95%CI: 2.38, 8.28), and Main Meals (6-week: 5.60 kcal/item, 95%CI: -11.17, 22.38; 6-month: 11.36, 95%CI: 0.83, 21.89). Results for all categories are in Supplementary Table 7.

Analysis of the energy content of items offered found only Breakfast to have a consistent decrease in energy per item (6-week, April 2022: -4.29 kcal/item, 95%CI: -6.33, -2.26; 6-month, April 2022: -3.60, 95%CI: -5.60, -1.60; 6-month, July 2022: -8.19, 95%CI: -12.86, -3.52). Other category results are shown in Supplementary Table 8.

No change was observed in overall retail sales, except drinks where there was an increase in low calorie items (6 weeks: 393 quantity increase, 95%CI: 135, 650; 6-month: 363 quantity increase, 95%CI: 1, 724). See Supplementary Table 9 and 10 for full results.

## Discussion

There was no evidence that the introduction of mandatory calorie-labelling in worksite canteens led to a decrease in the overall energy content of foods purchased, and no evidence of a substantive decrease in energy content of meals offered. While there was some evidence of a decrease in the latter, which may, over time, have led to a decrease in energy purchased, the effect was small in absolute terms, with the post-hoc analysis incorporating trends from a year before implementation finding an estimated reduction per week of -0.34 kcal per item. Sales of retail snacks, where no labelling changes occurred, were unchanged following implementation of calorie labelling on other items.

### Strengths and limitations

The strength of this study is the scale of this natural experiment, using data from 142 worksites in the primary analysis and 135 worksites in the post-hoc analysis, encompassing a range of business types, including offices and distribution or manufacturing centres. It is also, to our knowledge, one of the first studies to consider the implementation of calorie labelling policy in the United Kingdom using real-world purchase data.

The calorie-labelling legislation introduced in England required all businesses which sold food prepared for immediate consumption and with 250 or more employees, to introduce labels on a set date and timings meant we were unable to conduct a randomised controlled or stepped wedge trial. We therefore considered this as a natural experiment and used an interrupted time series design. However, ITS is a sensitive and complex analysis method and has some particular limitations [23]. For example, when seasonal menu changes were included as specific points in the model, some initial evidence for a seasonal effect was seen, as is evidenced by the -3.84 kcal/item decrease in energy purchased with the shift to the July 2022 menu (Fig. 1c). This finding is supported by well-researched seasonal variations in dietary patterns [3, 20–22, 24]. In a natural experiment, it is difficult to definitively conclude if this is the expected seasonal change in energy offered, or if it is something more. If data in this study included several years before and after the intervention, it may be possible to better account for these annually occurring seasonal dietary patterns, however, that was not possible.

In an effort to clarify some of the questions arising from the primary results, we conducted a post-hoc analysis. It indicated there was a significant, albeit small, effect on both energy purchased and offered over time following the implementation of labels. However, this post-hoc analysis also included data from 2021; a time when the UK had lockdowns related to the COVID-19 pandemic, potentially influencing both the type of food offered and purchased at sites, as well as which sites were open. This adds uncertainty to the reliability of these data as a true measure of typical seasonal trends.

Further, we did not have access to individual-level data, but only to cafeteria-level data. As a result, conclusions can only be drawn about the average energy content per item purchased, rather than the total energy content of all products each individual purchased. Given the high inflation rate and wider economic context following the COVID-19 pandemic, it is possible that changes in financial circumstance for individual employees may have impacted on their choices during this period in ways unconnected to the introduction of calorie labelling.

Finally, some research has shown that there is not perfect compliance with the calorie labelling legislation in the UK [18]. This catering provider included calorie information as part of an automated process, whereby calorie content should automatically be added to menu templates used by sites. The research team also received some photos during the initial implementation that showed calorie labels had been added to menus. However, the research team did not have site checks for all 142 sites or for the full duration of the post-implementation phase, so it is still possible that there were some days when labels were not implemented, for example, if the menu templates were not working.

### Implications

In the UK setting, randomised controlled trials have found no or minimal effects from the addition of energy labels to worksite cafeteria menus [25, 26]. This natural experiment complements those findings, suggesting that calorie labelling alone may not have a sizable impact on reducing the energy purchased, and therefore likely to be consumed, at a population level. Our study also found some, albeit limited, evidence consistent with menu renovation towards lower-calorie options, which has not been previously shown, though is consistent with indications from menu analysis in the USA following calorie labelling legislation [17]. This suggests that any evaluation of the impact from calorie labelling should consider changing business practices as well as directly influencing consumer behaviour.

Providing information to consumers can facilitate informed choices and is a popular policy option with the public, and potentially with policy makers, given its low intrusiveness [8, 11, 15], though it adds costs to businesses. These findings add to literature that shows calorie labels have no negative impact on consumption, and generally support evidence that the magnitude of the impact may increase over time, as has been observed with both New South Wales and United States labelling policies [14, 17]. This study, alongside previous research, provides insights into the potential impact that may be useful for other countries where calorie-labelling is being considered as a possible policy option.

## Conclusion

This natural experiment in worksite cafeterias suggests that adding calorie labels to menus did not lead to a reduction in energy purchased by customers. There was some evidence indicating impact may increase over time, perhaps attributable to changes in the calorie content of menu items, but the reduction was small, and cannot be directly attributed to an effect of calorie labelling.

### Supplementary Information

Supplementary Material 1

## Data Availability

This research was conducted using data from a nationwide catering provider. Access to the study dataset by external researchers is not permitted as this is defined as confidential information in the agreement. Access to the study data by external researchers will require the expressed written consent of the catering partner.

## References

[1] Bleich SN, Economos CD, Spiker ML, Vercammen KA, Vanepps EM, Block JP, Elbel B, Story M, Roberto CA. A systematic review of calorie labeling and modified calorie labeling interventions: impact on consumer and restaurant behavior. Obesity (Silver Spring). 2017;25:2018–44. 29045080 10.1002/oby.21940 PMC5752125

[2] Cantu-Jungles, TM, McCormack, LA, Slaven, JE, SlebodniK, M & Eicher-Miller, HA. A meta-analysis to determine the impact of restaurant menu labeling on calories and nutrients (ordered or consumed) in U.S. adults. Nutrients. 2017;9(10):1088. 10.3390/nu9101088 PMC5691705 28973989

[3] Capita R, Alonso-Calleja C. Differences in reported winter and summer dietary intakes in young adults in Spain. Int J Food Sci Nutr. 2005;56:431–43. 16361183 10.1080/09637480500407875

[4] Cawley J, Susskind A, Willage B. The impact of information disclosure on consumer behavior: evidence from a randomized field experiment of calorie labels on restaurant menus. J Policy Anal Manage. 2020;39:1020–42. 10.1002/pam.22219

[5] Crockett, RA, King, SE, Marteau, TM, Prevost, AT, Bignardi, G, Roberts, NW, Stubbs, B, Hollands, GJ & Jebb, SA. Nutritional labelling for healthier food or non‐alcoholic drink purchasing and consumption. Cochrane Database Syst Rev. 2018;2(2):CD009315. 10.1002/14651858.CD009315.pub2 PMC5846184 29482264

[6] Dai H, Alsalhe TA, Chalghaf N, Ricco M, Bragazzi NL, Wu J. The global burden of disease attributable to high body mass index in 195 countries and territories, 1990–2017: an analysis of the global burden of disease study. PLoS Med. 2020;17:e1003198. 10.1371/journal.pmed.1003198 PMC7386577 32722671

[7] Department of health and social care. New calorie labelling rules come into force to improve nation’s health. 2022. GOV.UK. https://www.gov.uk/government/news/new-calorie-labelling-rules-come-into-force-to-improve-nations-health.

[8] Diepeveen S, Ling T, Suhrcke M, Roland M, Marteau TM. Public acceptability of government intervention to change health-related behaviours: a systematic review and narrative synthesis. BMC Public Health. 2013;13:756. 23947336 10.1186/1471-2458-13-756 PMC3765153

[9] Ellison B, Lusk JL, Davis D. Looking at the label and beyond: the effects of calorie labels, health consciousness, and demographics on caloric intake in restaurants. Int J Behav Nutr Phys Act. 2013;10:21. 23394433 10.1186/1479-5868-10-21 PMC3598881

[10] Ellison B, Lusk JL, Davis D. The impact of restaurant calorie labels on food choice: results from a field experiment. Econ Inq. 2014;52:666–81. 10.1111/ecin.12069

[11] Hagmann D, Siegrist M, Hartmann C. Taxes, labels, or nudges? Public acceptance of various interventions designed to reduce sugar intake. Food Policy. 2018;79:156–65. 10.1016/j.foodpol.2018.06.008

[12] Long MW, Tobias DK, Cradock AL, Batchelder H, Gortmaker SL. Systematic review and meta-analysis of the impact of restaurant menu calorie labeling. Am J Public Health. 2015;105:e11–24. 25790388 10.2105/AJPH.2015.302570 PMC4386504

[13] NSW Food Authority. Evaluation of kilojoule menu labelling. NSW Food Authority. 2013. Available from: https://www.foodauthority.nsw.gov.au/sites/default/files/_Documents/scienceandtechnical/fastchoices_evaluation_report.pdf.

[14] Obesity Evidence Hub. Kilojoule labelling in fast food outlets [Online]. 2021. Available: https://www.obesityevidencehub.org.au/collections/prevention/kilojoule-labelling-in-fast-food-outlets. Accessed 1 Mar 2024.

[15] Pechey R, Reynolds JP, Cook B, Marteau TM, Jebb SA. Acceptability of policies to reduce consumption of red and processed meat: a population-based survey experiment. J Environ Psychol. 2022;81:101817. 10.1016/j.jenvp.2022.101817 PMC9742849 36523649

[16] Petimar J, Zhang F, Cleveland LP, Simon D, Gortmaker SL, Polacsek M, Bleich SN, Rimm EB, Roberto CA, Block JP. Estimating the effect of calorie menu labeling on calories purchased in a large restaurant franchise in the southern United States: quasi-experimental study. BMJ. 2019;367:l5837. 10.1136/bmj.l5837 PMC6818731 31666218

[17] Petimar J, Zhang F, Rimm EB, Simon D, Cleveland LP, Gortmaker SL, Bleich SN, Polacsek M, Roberto CA, Block JP. Changes in the calorie and nutrient content of purchased fast food meals after calorie menu labeling: a natural experiment. PLoS Med. 2021;18:e1003714. 10.1371/journal.pmed.1003714 PMC8312920 34252088

[18] Polden M, Jones A, Essman M, Adams J, Bishop T, Burgoine T, Donohue A, Sharp S, White M, Smith R, Robinson E. Point-of-choice kilocalorie labelling practices in large, out-of-home food businesses: a preobservational versus post observational study of labelling practices following implementation of The Calorie Labelling (Out of Home Sector) (England) Regulations 2021. BMJ Open. 2024;14:e080405. 10.1136/bmjopen-2023-080405 PMC11015320 38604637

[19] Robinson E, Marty L, Jones A, White M, Smith R, Adams J. Will calorie labels for food and drink served outside the home improve public health? BMJ. 2021;372:n40. 10.1136/bmj.n40 33472836

[20] Rossato SL, Olinto MT, Henn RL, Moreira LB, Camey SA, Anjos LA, Wahrlich V, Waissmann W, Fuchs FD, Fuchs SC. Seasonal variation in food intake and the interaction effects of sex and age among adults in southern Brazil. Eur J Clin Nutr. 2015;69:1015–22. 25828623 10.1038/ejcn.2015.22

[21] Stelmach-Mardas M, Kleiser C, Uzhova I, Penalvo JL, la Torre G, Palys W, Lojko D, Nimptsch K, Suwalska A, Linseisen J, Saulle R, Colamesta V, Boeing H. Seasonality of food groups and total energy intake: a systematic review and meta-analysis. Eur J Clin Nutr. 2016;70:700–8. 26757837 10.1038/ejcn.2015.224

[22] Sturm R, Patel D, Alexander E, Paramanund J. Seasonal cycles in food purchases and changes in BMI among South Africans participating in a health promotion programme. Public Health Nutr. 2016;19:2838–43. 27169872 10.1017/S1368980016000902 PMC10270870

[23] Turner SL, Karahalios A, Forbes AB, Taljaard M, Grimshaw JM, McKenzie JE. Comparison of six statistical methods for interrupted time series studies: empirical evaluation of 190 published series. BMC Med Res Methodol. 2021;21:134. 34174809 10.1186/s12874-021-01306-w PMC8235830

[24] van der Toorn JE, Cepeda M, Kiefte-de Jong JC, Franco OH, Voortman T, Schoufour JD. Seasonal variation of diet quality in a large middle-aged and elderly Dutch population-based cohort. Eur J Nutr. 2020;59:493–504. 30734846 10.1007/s00394-019-01918-5 PMC7058580

[25] Vasiljevic M, Cartwright E, Pilling M, Lee MM, Bignardi G, Pechey R, Hollands GJ, Jebb SA, Marteau TM. Impact of calorie labelling in worksite cafeterias: a stepped wedge randomised controlled pilot trial. Int J Behav Nutr Phys Act. 2018;15:41. 29754587 10.1186/s12966-018-0671-7 PMC5950179

[26] Vasiljevic M, Fuller G, Pilling M, Hollands GJ, Pechey R, Jebb SA, Marteau TM. What is the impact of increasing the prominence of calorie labelling? A stepped wedge randomised controlled pilot trial in worksite cafeterias. Appetite. 2019;141:104304. 10.1016/j.appet.2019.05.035 PMC8161726 31152762

[27] Wellard-Cole L, Goldsbury D, Havill M, Hughes C, Watson WL, Dunford EK, Chapman K. Monitoring the changes to the nutrient composition of fast foods following the introduction of menu labelling in New South Wales, Australia: an observational study. Public Health Nutr. 2018;21:1194–9. 29262878 10.1017/S1368980017003706 PMC10261402

[28] Zlatevska N, Neumann N, Dubelaar C. Mandatory calorie disclosure: a comprehensive analysis of its effect on consumers and retailers. J Retail. 2018;94:89–101. 10.1016/j.jretai.2017.09.007

