# Supplementary material for: The impact of the English calorie labelling policy on the energy content of food offered and purchased in worksite cafeterias: a natural experiment
Source: BMC Nutr. 2024 Aug 13;10:110. doi: 10.1186/s40795-024-00914-1 (PMC11321062; doi:10.1186/s40795-024-00914-1)
Supplement: Supplementary file 1 — Supplementary Material 1 [file 40795_2024_914_MOESM1_ESM.docx]

**Supplementary File A**

The model for analysis of the impact of calorie labelling implementation on energy purchased was as follows:

$$y=a+B_{1}I+ B_{2}t+ B_{3}I*t+B_{4}MenuChange+ \varepsilon$$

Where:

- $y$ is the outcome variable
- $a$ is the intercept
- $B$ are the regression coefficients
- $t$ is the time
- $I$ is a binary variable which is 0 before the implementation of the calorie labelling law and 1 after
- $MenuChange$ is a variable only included in the sensitivity analyses, that accounts for menu changes which occurred every 12 weeks

The model for analysis of the impact of calorie labelling implementation on menu composition was as follows:

$$y=a+B_{1}t+ B_{2}MenuChange+ \varepsilon$$

Where:

- $y$ is the outcome variable
- $a$ is the intercept
- $B$ are the regression coefficients
- $t$ is the time
- $MenuChange$ is a variable that accounts for menu changes which occurred every 12 weeks

The model for the post-hoc analysis was as follows:

$$y=a+B_{1}I+ B_{2}t+ B_{3}I*t+B_{4}month+ \varepsilon$$

Where:

- $y$ is the outcome variable
- $a$ is the intercept
- $B$ are the regression coefficients
- $t$ is the time
- $I$ is a binary variable which is 0 before the implementation of the calorie labelling law and 1 after
- $month$ is a variable which equates to a four-week period during the year (rather than a calendar month), with thirteen levels for the 52 weeks in the year, so that the same date in a given year has the same level for this variable

Supplementary Table 1. Models for energy purchased in the 6-week post-implementation analysis.

| Models: 6-week analysis |  | Coefficient | SE | p-value | Lower 95% CI | Upper 95% CI |
| --- | --- | --- | --- | --- | --- | --- |
| Energy per product purchased | Intercept | 240.08 | 0.91 | <0.001 | 238.31 | 241.86 |
|  | Time (in days) | -0.02 | 0.02 | 0.207 | -0.06 | 0.01 |
|  | Labelling Implementation | 0.60 | 1.61 | 0.707 | -2.54 | 3.75 |
|  | Labelling Implementation*time (in days) | -0.04 | 0.04 | 0.358 | -0.13 | 0.05 |
| Energy per product purchased (Breakfast) | Intercept | 155.41 | 0.54 | <0.001 | 154.35 | 156.47 |
|  | Time (in days) | -0.04 | 0.01 | <0.001 | -0.06 | -0.02 |
|  | Labelling Implementation | -2.40 | 0.94 | 0.010 | -4.24 | -0.56 |
|  | Labelling Implementation*time (in days) | 0.01 | 0.03 | 0.771 | -0.04 | 0.06 |
| Energy per product purchased (Cake/Pastries/Biscuits/  Discretionary) | Intercept | 354.33 | 0.98 | <0.001 | 352.40 | 356.25 |
|  | Time (in days) | 0.06 | 0.02 | 0.005 | 0.02 | 0.10 |
|  | Labelling Implementation | -6.65 | 1.03 | <0.001 | -8.68 | -4.62 |
|  | Labelling Implementation*time (in days) | 0.05 | 0.03 | 0.133 | -0.02 | 0.12 |
| Energy per product purchased (Fruit and Vegetables) | Intercept | 264.60 | 1.20 | <0.001 | 262.25 | 266.96 |
|  | Time (in days) | -0.08 | 0.03 | 0.006 | -0.14 | -0.02 |
|  | Labelling Implementation | 9.02 | 1.55 | <0.001 | 5.98 | 12.06 |
|  | Labelling Implementation*time (in days) | -0.13 | 0.04 | 0.001 | -0.20 | -0.05 |
| Energy per product purchased (Jacket Potatoes) | Intercept | 221.93 | 0.47 | <0.001 | 221.01 | 222.86 |
|  | Time (in days) | -0.02 | 0.01 | 0.089 | -0.04 | 0.00 |
|  | Labelling Implementation | 4.53 | 0.64 | <0.001 | 3.29 | 5.77 |
|  | Labelling Implementation*time (in days) | -0.05 | 0.02 | 0.017 | -0.08 | -0.01 |
| Energy per product purchased (Meals) | Intercept | 484.57 | 3.44 | <0.001 | 477.83 | 491.32 |
|  | Time (in days) | -0.17 | 0.07 | 0.015 | -0.31 | -0.03 |
|  | Labelling Implementation | 5.60 | 8.56 | 0.513 | -11.17 | 22.38 |
|  | Labelling Implementation*time (in days) | 0.27 | 0.38 | 0.477 | -0.47 | 1.01 |
| Energy per product purchased (Miscellaneous and Condiments) | Intercept | 102.89 | 0.77 | <0.001 | 101.37 | 104.40 |
|  | Time (in days) | 0.02 | 0.02 | 0.397 | -0.02 | 0.06 |
|  | Labelling Implementation | -0.38 | 1.87 | 0.837 | -4.04 | 3.27 |
|  | Labelling Implementation*time (in days) | -0.04 | 0.06 | 0.559 | -0.15 | 0.08 |
| Energy per product purchased (Salads and Cold Snacks) | Intercept | 148.75 | 1.06 | <0.001 | 146.67 | 150.83 |
|  | Time (in days) | -0.08 | 0.03 | 0.003 | -0.13 | -0.03 |
|  | Labelling Implementation | -1.61 | 1.82 | 0.376 | -5.18 | 1.96 |
|  | Labelling Implementation*time (in days) | 0.26 | 0.06 | <0.001 | 0.14 | 0.38 |
| Energy per product purchased (Sandwiches) | Intercept | 437.81 | 1.01 | <0.001 | 435.83 | 439.79 |
|  | Time (in days) | -0.01 | 0.02 | 0.671 | -0.06 | 0.04 |
|  | Labelling Implementation | -6.78 | 1.22 | <0.001 | -9.16 | -4.39 |
|  | Labelling Implementation*time (in days) | 0.08 | 0.03 | 0.013 | 0.02 | 0.15 |
| Energy per product purchased (Savoury Snacks) | Intercept | 455.57 | 3.25 | <0.001 | 449.19 | 461.94 |
|  | Time (in days) | 0.07 | 0.07 | 0.357 | -0.07 | 0.21 |
|  | Labelling Implementation | 1.14 | 3.99 | 0.775 | -6.68 | 8.96 |
|  | Labelling Implementation*time (in days) | -0.19 | 0.15 | 0.209 | -0.48 | 0.11 |
| Energy per product purchased (Starter) | Intercept | 113.74 | 0.76 | <0.001 | 112.26 | 115.22 |
|  | Time (in days) | -0.02 | 0.02 | 0.190 | -0.06 | 0.01 |
|  | Labelling Implementation | -0.17 | 1.17 | 0.887 | -2.46 | 2.13 |
|  | Labelling Implementation*time (in days) | -0.02 | 0.05 | 0.703 | -0.11 | 0.08 |

Supplementary Table 2. Models for energy purchased in the 6-month post-implementation analysis.

| Model 6-month Analysis |  | Coefficient | SE | p-value | Upper 95% | Lower 95% |
| --- | --- | --- | --- | --- | --- | --- |
| Energy per product purchased | Intercept | 240.08 | 0.91 | <0.001 | 238.31 | 241.86 |
|  | Time (in days) | -0.02 | 0.02 | 0.207 | -0.06 | 0.01 |
|  | Labelling Implementation | 1.60 | 1.31 | 0.220 | -0.96 | 4.16 |
|  | Labelling Implementation*time (in days) | -0.04 | 0.02 | 0.099 | -0.08 | 0.01 |
| Energy per product purchased (Breakfast) | Intercept | 960.97 | 183.64 | <0.001 | 601.04 | 1320.89 |
|  | Time (in days) | -0.04 | 0.01 | <0.001 | -0.06 | -0.02 |
|  | Labelling Implementation | -3.90 | 1.01 | <0.001 | -5.89 | -1.92 |
|  | Labelling Implementation*time (in days) | 0.04 | 0.01 | <0.001 | 0.02 | 0.06 |
| Energy per product purchased (Cake/Pastries/Biscuits/ Discretionary) | Intercept | -749.43 | 396.26 | 0.059 | -1526.08 | 27.21 |
|  | Time (in days) | 0.06 | 0.02 | 0.005 | 0.02 | 0.10 |
|  | Labelling Implementation | -3.17 | 1.86 | 0.087 | -6.81 | 0.46 |
|  | Labelling Implementation*time (in days) | -0.05 | 0.02 | 0.016 | -0.09 | -0.01 |
| Energy per product purchased (Fruit and Vegetables) | Intercept | 1773.89 | 551.44 | 0.001 | 693.10 | 2854.69 |
|  | Time (in days) | -0.08 | 0.03 | 0.006 | -0.14 | -0.02 |
|  | Labelling Implementation | 5.33 | 1.51 | <0.001 | 2.38 | 8.28 |
|  | Labelling Implementation*time (in days) | 0.10 | 0.03 | 0.001 | 0.04 | 0.17 |
| Energy per product purchased (Jacket Potatoes) | Intercept | 575.52 | 207.42 | 0.006 | 168.98 | 982.06 |
|  | Time (in days) | -0.02 | 0.01 | 0.089 | -0.04 | 0.00 |
|  | Labelling Implementation | 2.35 | 1.04 | 0.024 | 0.31 | 4.39 |
|  | Labelling Implementation*time (in days) | 0.03 | 0.01 | 0.009 | 0.01 | 0.05 |
| Energy per product purchased (Meals) | Intercept | 484.57 | 3.44 | <0.001 | 477.83 | 491.32 |
|  | Time (in days) | -0.17 | 0.07 | 0.015 | -0.31 | -0.03 |
|  | Labelling Implementation | 11.36 | 5.37 | 0.035 | 0.83 | 21.89 |
|  | Labelling Implementation*time (in days) | 0.06 | 0.08 | 0.467 | -0.10 | 0.21 |
| Energy per product purchased (Miscellaneous and Condiments) | Intercept | -222.36 | 383.42 | 0.562 | -973.86 | 529.14 |
|  | Time (in days) | 0.02 | 0.02 | 0.397 | -0.02 | 0.06 |
|  | Labelling Implementation | 0.31 | 0.98 | 0.754 | -1.61 | 2.22 |
|  | Labelling Implementation*time (in days) | -0.06 | 0.02 | 0.007 | -0.10 | -0.02 |
| Energy per product purchased (Salads and Cold Snacks) | Intercept | 1592.59 | 491.64 | 0.001 | 629.00 | 2556.17 |
|  | Time (in days) | -0.08 | 0.03 | 0.003 | -0.13 | -0.03 |
|  | Labelling Implementation | 1.21 | 1.55 | 0.438 | -1.84 | 4.25 |
|  | Labelling Implementation*time (in days) | 0.06 | 0.03 | 0.025 | 0.01 | 0.12 |
| Energy per product purchased (Sandwiches) | Intercept | 640.27 | 475.97 | 0.179 | -292.62 | 1573.16 |
|  | Time (in days) | -0.01 | 0.02 | 0.671 | -0.06 | 0.04 |
|  | Labelling Implementation | -6.03 | 1.10 | <0.001 | -8.19 | -3.87 |
|  | Labelling Implementation*time (in days) | 0.04 | 0.03 | 0.156 | -0.01 | 0.09 |
| Energy per product purchased (Savoury Snacks) | Intercept | -808.58 | 1370.78 | 0.555 | -3495.26 | 1878.09 |
|  | Time (in days) | 0.07 | 0.07 | 0.357 | -0.07 | 0.21 |
|  | Labelling Implementation | 0.89 | 3.68 | 0.808 | -6.32 | 8.11 |
|  | Labelling Implementation*time (in days) | -0.10 | 0.07 | 0.178 | -0.25 | 0.05 |
| Energy per product purchased (Starter) | Intercept | 579.35 | 354.32 | 0.102 | -115.12 | 1273.81 |
|  | Time (in days) | -0.02 | 0.02 | 0.190 | -0.06 | 0.01 |
|  | Labelling Implementation | -3.77 | 1.67 | 0.024 | -7.06 | -0.49 |
|  | Labelling Implementation*time (in days) | 0.06 | 0.02 | 0.004 | 0.02 | 0.10 |

Supplementary Table 3. Post-hoc analysis and sensitivity analyses for energy per product purchased.

| Models: Post-Hoc and Sensitivity |  | Coefficient | SE | p-value | Upper 95% | Lower 95% |
| --- | --- | --- | --- | --- | --- | --- |
| Energy per product purchased (post-hoc analysis) | Intercept | 230.25 | 0.79 | <0.001 | 228.67 | 231.83 |
|  | Time (in weeks) | -0.03 | 0.03 | 0.402 | -0.09 | 0.03 |
|  | Calorie Labelling Implementation | 3.01 | 1.50 | 0.050 | 0.43 | 5.60 |
|  | Labelling Implementation*time (in weeks) | -0.65 | 0.06 | <0.001 | -0.81 | -0.49 |
|  | First 4-week period | 0.71 | 0.25 | 0.007 | -0.31 | 1.74 |
|  | Second 4-week period | 5.76 | 0.52 | <0.001 | 3.36 | 8.16 |
|  | Third 4-week period | 6.90 | 1.75 | <0.001 | 4.23 | 9.57 |
|  | Fourth 4-week period | 6.10 | 1.34 | <0.001 | 3.74 | 8.46 |
|  | Fifth 4-week period | 7.79 | 1.25 | <0.001 | 4.46 | 11.12 |
|  | Sixth 4-week period | 7.92 | 1.14 | <0.001 | 5.61 | 10.24 |
|  | Seventh 4-week period | 3.72 | 0.36 | <0.001 | 2.32 | 5.12 |
|  | Eigth 4-week period | 3.89 | 0.39 | <0.001 | 2.26 | 5.52 |
|  | Ninth 4-week period | 1.18 | 0.45 | 0.012 | -1.08 | 3.45 |
|  | Tenth 4-week period | 3.16 | 0.52 | <0.001 | 2.00 | 4.33 |
|  | Eleventh 4-week period | 2.27 | 0.71 | 0.002 | 0.41 | 4.14 |
|  | Twelfth 4-week period | 3.27 | 0.72 | <0.001 | 1.81 | 4.73 |
|  |  |  |  |  |  |  |
| Energy per product purchased | Intercept | 225.98 | 0.68 | <0.001 | 224.61 | 227.34 |
| (post-hoc sensitivity analysis, n=97) | Time (in weeks) | -0.09 | 0.02 | <0.001 | -0.13 | -0.04 |
|  | Calorie Labelling Implementation | 3.10 | 0.93 | 0.002 | 1.23 | 4.97 |
|  | Labelling Implementation*time (in weeks) | -0.45 | 0.05 | <0.001 | -0.56 | -0.34 |
|  | First 4-week period | 1.33 | 0.42 | 0.003 | 0.48 | 2.18 |
|  | Second 4-week period | 4.11 | 0.56 | <0.001 | 2.98 | 5.23 |
|  | Third 4-week period | 2.49 | 0.69 | 0.001 | 1.11 | 3.88 |
|  | Fourth 4-week period | 1.71 | 0.78 | 0.034 | 0.14 | 3.29 |
|  | Fifth 4-week period | 3.67 | 1.90 | 0.058 | -0.13 | 7.48 |
|  | Sixth 4-week period | 4.12 | 0.81 | <0.001 | 2.50 | 5.73 |
|  | Seventh 4-week period | 4.95 | 0.70 | <0.001 | 3.55 | 6.36 |
|  | Eighth 4-week period | 5.81 | 0.82 | <0.001 | 4.16 | 7.46 |
|  | Ninth 4-week period | 3.97 | 0.84 | <0.001 | 2.28 | 5.66 |
|  | Tenth 4-week period | 3.76 | 0.52 | <0.001 | 2.72 | 4.80 |
|  | Eleventh 4-week period | 2.59 | 0.75 | 0.001 | 1.08 | 4.10 |
|  | Twelfth 4-week period | 3.67 | 0.68 | <0.001 | 2.30 | 5.03 |
|  |  |  |  |  |  |  |
| Energy per product purchased (n = 137, 6-week analysis) | Intercept | 237.45 | 0.76 | <0.001 | 235.95 | 238.94 |
|  | Time (in days) | -0.05 | 0.02 | 0.001 | -0.08 | -0.02 |
|  | Labelling Implementation | 0.71 | 1.38 | 0.610 | -2.00 | 3.41 |
|  | Labelling Implementation*time (in days) | 0.02 | 0.04 | 0.696 | -0.06 | 0.10 |
|  |  |  |  |  |  |  |
| Energy per product purchased (n = 137, 6-month analysis) | Intercept | 237.45 | 0.76 | <0.001 | 235.95 | 238.94 |
|  | Time (in days) | -0.05 | 0.02 | 0.001 | -0.08 | -0.02 |
|  | Labelling Implementation | 2.51 | 1.36 | 0.066 | -0.17 | 5.18 |
|  | Labelling Implementation*time (in days) | 0.00 | 0.02 | 0.865 | -0.04 | 0.04 |
|  |  |  |  |  |  |  |
| Energy per product purchased (with seasonal menu change) | Intercept | 683.79 | 350.56 | 0.053 | -7.87 | 1375.45 |
|  | Time (in days) | -0.02 | 0.02 | 0.208 | -0.06 | 0.01 |
|  | Labelling Implementation | 0.73 | 1.21 | 0.547 | -1.66 | 3.11 |
|  | Labelling Implementation*time (in days) | 0.00 | 0.02 | 0.840 | -0.05 | 0.04 |
|  | July 2022 menu change | -3.84 | 1.28 | 0.003 | -6.36 | -1.33 |

Supplementary Table 4. Models for mean energy per item offered in the 6-week post-implementation analysis.

| Models: 6-week analysis |  | Coefficient | SE | p-value | Lower 95% CI | Upper 95% CI |
| --- | --- | --- | --- | --- | --- | --- |
| Mean kcal per menu items offered | Intercept | 295.13 | 261.54 | 0.262 | -224.96 | 815.22 |
|  | Time (in days) | -0.001 | 0.01 | 0.946 | -0.03 | 0.03 |
|  | April 2022 menu change (at time of labelling implementation) | -2.03 | 1.12 | 0.074 | -4.26 | 0.20 |
| Mean kcal per menu items offered (Breakfast) | Intercept | 14.53 | 212.25 | 0.946 | -407.56 | 436.61 |
|  | Time (in days) | 0.01 | 0.01 | 0.430 | -0.01 | 0.03 |
|  | April 2022 menu change (at time of labelling implementation) | -4.29 | 1.02 | <0.001 | -6.33 | -2.26 |
| Mean kcal per menu items offered (Cake/Pastries/Biscuits/Discretionary) | Intercept | -1205.52 | 354.51 | 0.001 | -1910.49 | -500.55 |
|  | Time (in days) | 0.08 | 0.02 | <0.001 | 0.04 | 0.12 |
|  | April 2022 menu change (at time of labelling implementation) | -5.99 | 1.33 | <0.001 | -8.64 | -3.34 |
| Mean kcal per menu items offered (Fruit and Vegetables) | Intercept | 2902.33 | 445.75 | <0.001 | 2015.91 | 3788.76 |
|  | Time (in days) | -0.14 | 0.02 | <0.001 | -0.19 | -0.10 |
|  | April 2022 menu change (at time of labelling implementation) | 3.99 | 1.52 | 0.010 | 0.96 | 7.02 |
| Mean kcal per menu items offered (Jacket Potatoes) | Intercept | 633.01 | 226.93 | 0.007 | 181.74 | 1084.28 |
|  | Time (in days) | -0.02 | 0.01 | 0.059 | -0.05 | 0.00 |
|  | April 2022 menu change (at time of labelling implementation) | 2.62 | 0.85 | 0.003 | 0.93 | 4.31 |
| Mean kcal per menu items offered (Meals) | Intercept | 2604.36 | 967.24 | 0.009 | 680.90 | 4527.81 |
|  | Time (in days) | -0.11 | 0.05 | 0.032 | -0.21 | -0.01 |
|  | April 2022 menu change (at time of labelling implementation) | 1.50 | 3.90 | 0.701 | -6.25 | 9.25 |
| Mean kcal per menu items offered (Miscellaneous/ Condiments) | Intercept | -227.98 | 392.15 | 0.563 | -1007.82 | 551.86 |
|  | Time (in days) | 0.02 | 0.02 | 0.391 | -0.02 | 0.06 |
|  | April 2022 menu change (at time of labelling implementation) | -0.76 | 1.26 | 0.549 | -3.27 | 1.75 |
| Mean kcal per menu items offered (Salads and Cold Snacks) | Intercept | 1343.93 | 283.53 | <0.001 | 780.11 | 1907.76 |
|  | Time (in days) | -0.06 | 0.01 | <0.001 | -0.09 | -0.03 |
|  | April 2022 menu change (at time of labelling implementation) | 0.85 | 1.04 | 0.414 | -1.22 | 2.92 |
| Mean kcal per menu items offered (Sandwiches) | Intercept | 515.25 | 347.49 | 0.142 | -175.77 | 1206.27 |
|  | Time (in days) | -0.004 | 0.02 | 0.813 | -0.04 | 0.03 |
|  | April 2022 menu change (at time of labelling implementation) | -1.86 | 1.36 | 0.175 | -4.56 | 0.84 |
| Mean kcal per menu items offered (Savoury Snacks) | Intercept | -101.08 | 1033.01 | 0.922 | -2155.33 | 1953.17 |
|  | Time (in days) | 0.03 | 0.05 | 0.587 | -0.08 | 0.14 |
|  | April 2022 menu change (at time of labelling implementation) | 0.32 | 2.89 | 0.913 | -5.43 | 6.07 |
| Mean kcal per menu items offered (Starters) | Intercept | 293.60 | 355.28 | 0.411 | -412.90 | 1000.11 |
|  | Time (in days) | -0.01 | 0.02 | 0.618 | -0.05 | 0.03 |
|  | April 2022 menu change (at time of labelling implementation) | -2.01 | 1.21 | 0.100 | -4.42 | 0.39 |

Supplementary Table 5. Models for mean energy per item offered in the 6-month post-implementation analysis.

| Models: 6-month analysis | | Coefficient | SE | p-value | | Lower 95% CI | Upper 95% CI |
| --- | --- | --- | --- | --- | --- | --- | --- |
| Intercept | Mean kcal per menu items offered | 451.99 | 163.12 | 0.006 | 130.16 | | 773.83 |
| Time (in days) |  | -0.01 | 0.01 | 0.286 | -0.03 | | 0.01 |
| April 2022 menu change (at time of labelling implementation) |  | -1.79 | 0.83 | 0.032 | -3.42 | | -0.15 |
| July 2022 menu change |  | -4.19 | 1.75 | 0.018 | -7.65 | | -0.73 |
| Intercept | Mean kcal per menu items offered (Breakfast) | 284.57 | 200.52 | 0.158 | -111.05 | | 680.18 |
| Time (in days) |  | -0.01 | 0.01 | 0.613 | -0.03 | | 0.02 |
| April 2022 menu change (at time of labelling implementation) |  | -3.60 | 1.01 | 0.000 | -5.60 | | -1.60 |
| July 2022 menu change |  | -8.19 | 2.37 | 0.001 | -12.86 | | -3.52 |
| Intercept | Mean kcal per menu items offered (Cake/ Pastries /Biscuits/ Discretionary) | -318.36 | 333.81 | 0.341 | -976.94 | | 340.22 |
| Time (in days) |  | 0.03 | 0.02 | 0.051 | 0.00 | | 0.07 |
| April 2022 menu change (at time of labelling implementation) |  | -2.64 | 1.91 | 0.169 | -6.42 | | 1.14 |
| July 2022 menu change |  | -7.38 | 3.88 | 0.058 | -15.03 | | 0.27 |
| Intercept | Mean kcal per menu items offered (Fruit and Vegetables) | 634.63 | 453.02 | 0.163 | -259.16 | | 1528.42 |
| Time (in days) |  | -0.02 | 0.02 | 0.346 | -0.07 | | 0.02 |
| April 2022 menu change (at time of labelling implementation) |  | -1.82 | 3.04 | 0.551 | -7.82 | | 4.18 |
| July 2022 menu change |  | 2.34 | 5.00 | 0.640 | -7.52 | | 12.20 |
| Intercept | Mean kcal per menu items offered (Jacket Potatoes) | 348.40 | 185.13 | 0.061 | -16.85 | | 713.65 |
| Time (in days) |  | -0.01 | 0.01 | 0.421 | -0.03 | | 0.01 |
| April 2022 menu change (at time of labelling implementation) |  | 1.22 | 0.92 | 0.185 | -0.59 | | 3.04 |
| July 2022 menu change |  | 4.00 | 2.03 | 0.050 | -0.01 | | 8.01 |
| Intercept | Mean kcal per menu items offered (Meals) | 1767.96 | 550.94 | 0.002 | 680.98 | | 2854.93 |
| Time (in days) |  | -0.07 | 0.03 | 0.021 | -0.12 | | -0.01 |
| April 2022 menu change (at time of labelling implementation) |  | 0.18 | 3.75 | 0.961 | -7.22 | | 7.59 |
| July 2022 menu change |  | -2.22 | 5.55 | 0.689 | -13.17 | | 8.72 |
| Intercept | Mean kcal per menu items offered (Miscellaneous and Condiments) | 542.13 | 346.72 | 0.120 | -141.93 | | 1226.18 |
| Time (in days) |  | -0.02 | 0.02 | 0.213 | -0.06 | | 0.01 |
| April 2022 menu change (at time of labelling implementation) |  | 0.19 | 1.45 | 0.895 | -2.66 | | 3.04 |
| July 2022 menu change |  | 0.14 | 3.03 | 0.964 | -5.84 | | 6.11 |
| Intercept | Mean kcal per menu items offered (Salads and Cold Snacks) | 1075.14 | 161.59 | 0.000 | 756.33 | | 1393.95 |
| Time (in days) |  | -0.05 | 0.01 | 0.000 | -0.07 | | -0.03 |
| April 2022 menu change (at time of labelling implementation) |  | -0.20 | 0.76 | 0.796 | -1.70 | | 1.30 |
| July 2022 menu change |  | 2.74 | 1.50 | 0.070 | -0.22 | | 5.70 |
| Intercept | Mean kcal per menu items offered (Sandwiches) | 355.08 | 219.39 | 0.107 | -77.76 | | 787.92 |
| Time (in days) |  | 0.00 | 0.01 | 0.723 | -0.02 | | 0.03 |
| April 2022 menu change (at time of labelling implementation) |  | -2.98 | 1.59 | 0.062 | -6.11 | | 0.15 |
| July 2022 menu change |  | -2.75 | 2.40 | 0.254 | -7.48 | | 1.99 |
| Intercept | Mean kcal per menu items offered (Savoury Snacks) | -478.17 | 463.07 | 0.303 | -1391.79 | | 435.44 |
| Time (in days) |  | 0.05 | 0.02 | 0.044 | 0.00 | | 0.10 |
| April 2022 menu change (at time of labelling implementation) |  | -0.56 | 2.14 | 0.794 | -4.79 | | 3.67 |
| July 2022 menu change |  | -4.05 | 3.97 | 0.310 | -11.88 | | 3.79 |
| Intercept | Mean kcal per menu items offered (Starter) | -15.38 | 244.66 | 0.950 | -498.09 | | 467.33 |
| Time (in days) |  | 0.01 | 0.01 | 0.592 | -0.02 | | 0.03 |
| April 2022 menu change (at time of labelling implementation) |  | -4.04 | 1.63 | 0.014 | -7.27 | | -0.82 |
| July 2022 menu change |  | -0.65 | 2.75 | 0.814 | -6.08 | | 4.78 |

Supplementary Table 6. Post-hoc analysis and sensitivity analyses for mean energy per menu item offered.

| Models: Post-Hoc and Sensitivity |  | Coefficient | SE | p-value | Lower 95% CI | Upper 95% CI |
| --- | --- | --- | --- | --- | --- | --- |
| Mean kcal per menu items offered (post-hoc analysis) | Intercept | 290.17 | 0.94 | <0.001 | 288.33 | 292.00 |
|  | Time (in weeks) | -0.12 | 0.02 | <0.001 | -0.16 | -0.07 |
|  | Calorie Labelling Implementation | 3.39 | 1.09 | 0.002 | 1.26 | 5.52 |
|  | Labelling Implementation* time (in weeks) | -0.34 | 0.07 | <0.001 | -0.48 | -0.20 |
|  | First 4-week period | 0.48 | 1.24 | 0.702 | -1.96 | 2.91 |
|  | Second 4-week period | 1.42 | 1.79 | 0.426 | -2.08 | 4.92 |
|  | Third 4-week period | 0.20 | 1.04 | 0.847 | -1.83 | 2.24 |
|  | Fourth 4-week period | 0.29 | 1.51 | 0.846 | -2.67 | 3.26 |
|  | Fifth 4-week period | 1.42 | 1.35 | 0.290 | -1.21 | 4.06 |
|  | Sixth 4-week period | 2.36 | 1.29 | 0.068 | -0.17 | 4.89 |
|  | Seventh 4-week period | -3.17 | 0.80 | <0.001 | -4.73 | -1.60 |
|  | Eighth 4-week period | -2.27 | 1.13 | 0.045 | -4.50 | -0.05 |
|  | Ninth 4-week period | -2.97 | 1.07 | 0.006 | -5.06 | -0.87 |
|  | Tenth 4-week period | -1.70 | 0.82 | 0.038 | -3.31 | -0.10 |
|  | Eleventh 4-week period | -1.11 | 1.21 | 0.360 | -3.49 | 1.27 |
|  | Twelfth 4-week period | 1.19 | 1.03 | 0.248 | -0.83 | 3.20 |
|  |  |  |  |  |  |  |
| Mean kcal per menu items offered | Intercept | 291.65 | 1.13 | <0.001 | 289.43 | 293.86 |
| (post-hoc sensitivity analysis, n = 97) | Time (in weeks) | -0.10 | 0.03 | <0.001 | -0.15 | -0.05 |
|  | Calorie Labelling Implementation | 2.65 | 1.29 | 0.040 | 0.12 | 5.18 |
|  | Labelling Implementation* time (in weeks) | -0.33 | 0.07 | <0.001 | -0.47 | -0.20 |
|  | First 4-week period | 2.62 | 1.03 | 0.011 | 0.60 | 4.63 |
|  | Second 4-week period | 1.75 | 1.48 | 0.238 | -1.16 | 4.66 |
|  | Third 4-week period | -0.05 | 0.78 | 0.949 | -1.58 | 1.48 |
|  | Fourth 4-week period | -0.70 | 1.23 | 0.571 | -3.12 | 1.72 |
|  | Fifth 4-week period | 1.08 | 1.40 | 0.441 | -1.67 | 3.84 |
|  | Sixth 4-week period | 3.73 | 1.14 | 0.001 | 1.49 | 5.96 |
|  | Seventh 4-week period | 1.72 | 0.82 | 0.035 | 0.12 | 3.32 |
|  | Eighth 4-week period | 2.36 | 1.22 | 0.052 | -0.02 | 4.75 |
|  | Ninth 4-week period | 1.26 | 1.21 | 0.299 | -1.11 | 3.63 |
|  | Tenth 4-week period | -1.19 | 0.95 | 0.211 | -3.06 | 0.67 |
|  | Eleventh 4-week period | -0.02 | 1.20 | 0.990 | -2.37 | 2.34 |
|  | Twelfth 4-week period | 1.89 | 1.19 | 0.111 | -0.44 | 4.22 |
|  |  |  |  |  |  |  |
| Mean kcal per menu items offered (interrupted time series) | Intercept | 276.57 | 0.67 | <0.001 | 275.23 | 277.90 |
| (n=137, 6-week analysis) | Time (in days) | -0.01 | 0.01 | 0.348 | -0.04 | 0.01 |
|  | April 2022 menu change (at time of labelling implementation) | -1.58 | 1.03 | 0.126 | -3.62 | 0.46 |
|  |  |  |  |  |  |  |
| Mean kcal per menu items offered (interrupted time series) | Intercept | 276.56 | 0.47 | <0.001 | 275.62 | 277.50 |
| (n=137, 6-month analysis) | Time (in days) | -0.01 | 0.01 | 0.125 | -0.03 | 0.00 |
|  | April 2022 menu change (at time of labelling implementation) | -1.67 | 0.81 | 0.042 | -3.27 | -0.06 |
|  | July 2022 menu change | -3.95 | 1.70 | 0.021 | -7.31 | -0.60 |
|  |  |  |  |  |  |  |
| Mean kcal per menu items offered (interrupted time series, 6-week) | Intercept | 277.51 | 0.66 | <0.001 | 276.21 | 278.81 |
|  | Time (in days) | 0.002 | 0.01 | 0.858 | -0.02 | 0.03 |
|  | Labelling Implementation | -1.61 | 1.35 | 0.234 | -4.25 | 1.04 |
|  | Labelling Implementation* time (in days) | -0.03 | 0.04 | 0.418 | -0.10 | 0.04 |

Supplementary Table 7. Coefficients for the impact of the implementation by food category for energy per item purchased, using 6 weeks and 6 months following the implementation date for analysis, where *p<0.05, **p<0.01, ***p<0.001. No corrections for multiple comparisons were made in calculating these p-values or significance levels.

| **Food Category** | **6-week analysis** | **6-month analysis** |
| --- | --- | --- |
| Meals | 5.60 (-11.17, 22.38) | 11.36 (0.83, 21.89)* |
| Breakfast | -2.40 (-4.24, -0.56)* | -3.90 (-5.89, -1.92)*** |
| Salads and Cold Snacks | -6.65 (-8.68, -4.62)** | 1.21 (-1.84, 4.25) |
| Cakes Pastries Biscuits and Discretionary | -6.65 (-8.68, -4.62)** | -3.17 (-6.81, 0.46) |
| Fruits and Vegetables | 9.02 (5.98, 12.06)*** | 5.33 (2.38, 8.28)*** |
| Jacket Potatoes | 4.52 (3.29, 5.77)*** | 2.35 (0.31, 4.39)* |
| Miscellaneous/Condiments | -0.38 (-4.04, 3.27) | -0.06 (-0.10, -0.02)** |
| Starter | -0.17 (-2.46, 2.13) | -3.77 (-7.05, -0.49)* |
| Sandwiches | -6.78 (-9.16, -4.39)*** | -6.03 (-8.19, -3.87)*** |
| Savoury Snacks | 1.14 (-6.68, 8.96) | 0.89 (-6.32, 8.11) |

Supplementary Table 8. Changes in energy offered on menus by food category with the implementation (April 2022) and summer menu change (July 2022), where *p<0.05, **p<0.01, ***p<0.001. No corrections for multiple comparisons were made in calculating these p-values or significance levels.

| **Food Category** | **6-week analysis (April 2022)** | **6-month analysis (April 2022)** | **6-month analysis (July 2022)** |
| --- | --- | --- | --- |
| Meals | 1.50 (-6.24, 9.25) | 0.18 (-7.22, 7.59) | -2.22 (-13.17, 8.72) |
| Breakfast | -4.29 (-6.33, -2.26)*** | -3.60 (-5.60, -1.60)*** | -8.19 (-12.86, -3.52)*** |
| Salads and Cold Snacks | 0.85 (-1.22, 2.92) | -0.20 (-1.70, 1.30) | 2.74 (-0.22, 5.70) |
| Cakes Pastries Biscuits and Discretionary | -5.99 (-8.64, -3.34)*** | -2.64 (-6.42, 1.14) | -7.38 (-15.03, 0.27) |
| Fruits and Vegetables | 3.99 (0.96, 7.02)* | -1.82 (-7.82, 4.18) | 2.34 (-7.52, 12.20) |
| Jacket Potatoes | 2.62 (0.93, 4.31)** | 1.22 (-0.59, 3.04) | 4.00 (-0.01, 8.01) |
| Miscellaneous/Condiments | -0.76 (-3.27, 1.75) | 0.19 (-2.66, 3.04) | 0.14 (-5.84, 6.11) |
| Starter | -2.01 (-4.42, 0.39) | -4.04 (-7.26, -0.82)* | -0.65 (-6.08, 4.78) |
| Sandwiches | -1.86 (-4.56, 0.84) | -2.98 (-6.11, 0.15) | -2.75 (-7.48, 1.99) |
| Savoury Snacks | 0.32 (-5.43, 6.07) | -0.56 (-4.79, 3.67) | -4.04 (-11.88, 3.79) |

Supplementary Table 9. Retail snacks (i.e. pre-packaged crisps, chocolates, and snacks) and drinks quantity sales change at the implementation, using 6 weeks and 6 months following the implementation date for analysis, where *p<0.05, **p<0.01, ***p<0.001. No corrections for multiple comparisons were made in calculating these p-values or significance levels.

|  | **6-week analysis** | **6-month analysis** |
| --- | --- | --- |
| Retail Snackss | 148 (-146, 443) | -263 (-683, 157) |
| Sales of Sugar-Sweetened Drinks | 393 (135, 650)** | 489 (-56, 1034) |
| Sales of Low-Calorie Drinks | -150 (-319, 19) | 363 (1, 724)* |

Supplementary Table 10. Models for retail snacks and drink sales.

| Models: Retail and Drink Sales |  | Coefficient | SE | p-value | Lower 95% CI | Upper 95% CI |
| --- | --- | --- | --- | --- | --- | --- |
| Retail Snacks (6-week analysis) | Intercept | 13871 | 57552.0 | 0.810 | -98929 | 126670 |
|  | Total Quantity (all products) | 0 | 0.0 | <0.001 | 0 | 0 |
|  | Time (in days) | -1 | 3.0 | 0.759 | -7 | 5 |
|  | Labelling Intervention | 148 | 150.2 | 0.324 | -146 | 443 |
|  | Labelling Intervention*time (in days) | -23 | 5.3 | <0.001 | -33 | -12 |
| Sales of Low Calorie Drinks (6-week) | Intercept | -163236 | 30458.9 | <0.001 | -222934 | -103537 |
|  | Total Quantity (all products) | 0 | 0.0 | <0.001 | 0 | 0 |
|  | Time (in days) | 9 | 1.6 | <0.001 | 5 | 12 |
|  | Labelling Intervention | -150 | 86.3 | 0.083 | -319 | 19 |
|  | Labelling Intervention*time (in days) | 7 | 2.4 | 0.005 | 2 | 11 |
| Sales of Sugar Sweetened Beverages (6-week) | Intercept | 80139 | 41712.1 | 0.055 | -1615 | 161893 |
|  | Total Quantity (all products) | 0 | 0.0 | <0.001 | 0 | 0 |
|  | Time (in days) | -4 | 2.2 | 0.058 | -8 | 0 |
|  | Labelling Intervention | 393 | 131.2 | 0.003 | 135 | 650 |
|  | Labelling Intervention*time (in days) | 1 | 3.1 | 0.636 | -5 | 7 |
| Retail Snacks (6-month analysis) | Intercept | -6472 | 59077.4 | 0.913 | -122261 | 109318 |
|  | Total Quantity (all products) | 0 | 0.0 | <0.001 | 0 | 0 |
|  | Time (in days) | 0 | 3.1 | 0.955 | -6 | 6 |
|  | Labelling Intervention | -263 | 214.4 | 0.220 | -683 | 157 |
|  | Labelling Intervention*time (in days) | -3 | 3.0 | 0.305 | -9 | 3 |
| Sales of Low Calorie Drinks (6-month) | Intercept | -154460 | 31133.4 | <0.001 | -215481 | -93440 |
|  | Total Quantity (all products) | 0 | 0.0 | <0.001 | 0 | 0 |
|  | Time (in days) | 8 | 1.6 | <0.001 | 5 | 11 |
|  | Labelling Intervention | 363 | 184.6 | 0.049 | 1 | 724 |
|  | Labelling Intervention*time (in days) | -7 | 3.3 | 0.036 | -13 | 0 |
| Sales of Sugar Sweetened Beverages (6-month) | Intercept | 56730 | 42299.0 | 0.180 | -26175 | 139634 |
|  | Total Quantity (all products) | 0 | 0.0 | <0.001 | 0 | 0 |
|  | Time (in days) | -3 | 2.2 | 0.196 | -7 | 1 |
|  | Labelling Intervention | 489 | 278.1 | 0.079 | -56 | 1034 |
|  | Labelling Intervention*time (in days) | 7 | 4.3 | 0.131 | -2 | 15 |
